# Loss of the neuroprotective factor Sphingosine 1-phosphate early in Alzheimer’s disease pathogenesis

**DOI:** 10.1186/2051-5960-2-9

**Published:** 2014-01-23

**Authors:** Timothy A Couttas, Nupur Kain, Benjamin Daniels, Xin Ying Lim, Claire Shepherd, Jillian Kril, Russell Pickford, Hongyun Li, Brett Garner, Anthony S Don

**Affiliations:** 1 Prince of Wales Clinical School, Faculty of Medicine, University of New South Wales, level 2, C25 Lowy Building, Sydney 2052, NSW, Australia; 2 Bioanalytical Mass Spectrometry Facility, University of New South Wales, Sydney 2052, NSW, Australia; 3 Neuroscience Research Australia, Sydney 2031, NSW, Australia; 4 Disciplines of Medicine and Pathology, Sydney Medical School, The University of Sydney, Sydney 2006, NSW, Australia; 5 Illawarra Health and Medical Research Institute, School of Biological Sciences, University of Wollongong, Wollongong, NSW 2522, Australia

**Keywords:** Sphingosine 1-phosphate, Sphingosine kinase, Sphingolipid, Alzheimer’s disease, Apolipoprotein E

## Abstract

**Background:**

The greatest genetic risk factor for late-onset Alzheimer's disease (AD) is the ϵ4 allele of Apolipoprotein E (ApoE). ApoE regulates secretion of the potent neuroprotective signaling lipid Sphingosine 1-phosphate (S1P). S1P is derived by phosphorylation of sphingosine, catalysed by sphingosine kinases 1 and 2 (SphK1 and 2), and SphK1 positively regulates glutamate secretion and synaptic strength in hippocampal neurons. S1P and its receptor family have been subject to intense pharmacological interest in recent years, following approval of the immunomodulatory drug Fingolimod, an S1P mimetic, for relapsing multiple sclerosis.

**Results:**

We quantified S1P levels in six brain regions that are differentially affected by AD pathology, in a cohort of 34 post-mortem brains, divided into four groups based on Braak neurofibrillary tangle staging. S1P declined with increasing Braak stage, and this was most pronounced in brain regions most heavily affected by AD pathology. The S1P/sphingosine ratio was 66% and 64% lower in Braak stage III/IV hippocampus (p = 0.010) and inferior temporal cortex (p = 0.014), respectively, compared to controls. In accordance with this change, both SphK1 and SphK2 activity declined with increasing Braak pathology in the hippocampus (p = 0.032 and 0.047, respectively). S1P/sphingosine ratio was 2.5-fold higher in hippocampus of ApoE2 carriers compared to ApoE4 carriers, and multivariate regression showed a significant association between APOE genotype and hippocampal S1P/sphingosine (p = 0.0495), suggesting a new link between APOE genotype and pre-disposition to AD.

**Conclusions:**

This study demonstrates loss of S1P and sphingosine kinase activity early in AD pathogenesis, and prior to AD diagnosis. Our findings establish a rationale for further exploring S1P receptor pharmacology in the context of AD therapy.

## Background

Alzheimer’s disease (AD) is the most common form of dementia, currently estimated to afflict 25–30 million people worldwide, with the number of patients diagnosed expected to double by 2030 [1,2]. The neuropathological hallmarks that characterise AD include (i) synaptic loss, which correlates with cognitive decline; (ii) aggregates of amyloid-β peptides (Aβ) generated by cleavage of the Amyloid Precursor Protein (APP); (iii) neurofibrillary tangles (NFTs) comprised of hyperphosphorylated tau protein, and (iv) gliosis and neuroinflammation [3-7]. These pathological features result in neuronal loss and brain atrophy as the disease progresses [3,7,8]. Results from clinical trials aimed at reducing Aβ burden indicate that by the time patients are diagnosed with clinical AD the likelihood of successful treatment has greatly diminished [3,9]. These results underscore the importance of further research into the pathological changes that precede neuronal loss and cognitive impairment, with the ultimate aim of identifying biomarkers of early disease pathogenesis and new targets for therapeutic intervention.

Over 99% of AD cases are the age-related, late onset form of the disease, for which the greatest genetic risk factor is the APOE ϵ4 allele. Apolipoprotein E (ApoE) is a major lipid transport protein of the central nervous system (CNS) that also mediates the transport and clearance of Aβ, reviewed in [10,11]. Homozygous carriers of the ϵ4 allele have a 12-fold increased risk of developing AD, compared with non-carriers [12]. Conversely, the ϵ2 allele of the APOE gene has shown protective effects against AD [13]. Genetic variants of clusterin/Apolipoprotein J [14,15] and the lipid transporter ABCA7 [16] also confer increased risk for late-onset AD, implicating altered lipid homeostasis in AD pathogenesis.

Sphingosine 1-phosphate (S1P) is a potent lipid signalling molecule that associates with ApoE in high density lipoprotein (HDL) complexes in the CNS [17,18]. S1P is formed by phosphorylation of the membrane lipid sphingosine, a reaction that is catalysed by sphingosine kinase 1 (SphK1) or 2 (SphK2). The S1P formed may then be secreted, and signals with nanomolar potency through a family of five G-protein coupled receptors, S1P_1 – 5_, that are specific for S1P [19,20]. Thus, S1P may signal in an autocrine fashion, feeding back on the cell of origin, or a paracrine fashion by affecting other cell types in the local microenvironment. S1P and its receptor signalling pathways have been implicated in a wide array of physiological and cellular processes, including in the CNS. S1P is essential for development of the neural tube and vascular system during embryogenesis [21]. It is a potent cytoprotective factor [22] that has been shown to protect cultured cortical neurons against Aβ toxicity [23]. Signalling through pre-synaptic S1P_3_ receptors, S1P also stimulates glutamate secretion in hippocampal neurons, promoting long-term potentiation and memory consolidation [24,25]. Similarly SphK1 localised to pre-synaptic terminals is required for neurotransmitter release and charge transfer in response to acetylcholine stimulation [26].

S1P and its receptors have been shown to be a good target for pharmacological intervention in neurology, with the recent approval of the immunosuppressive sphingosine analogue FTY720/Fingolimod™ as a front-line oral therapeutic for the treatment of relapsing multiple sclerosis [27,28]. FTY720 is a synthetic analogue of sphingosine that is rapidly phosphorylated by SphK2 *in vivo*, forming the S1P mimetic FTY720-phosphate, which is a highly potent agonist of receptors S1P_1_, S1P_3_, S1P_4_ and S1P_5_[27,29]. FTY720-phosphate binding to S1P_1_ on lymphocytes and/or lymph node endothelial cells inhibits lymphocyte egress from the lymph nodes and thymus into the blood stream, thereby suppressing the adaptive immune system and autoimmune responses [20,30]. FTY720 also accumulates in the CNS, and its administration in experimental autoimmune encephalomyelitis, an animal model of multiple sclerosis, did not reduce disease symptoms in mice that lacked S1P_1_ expression in astrocytes, indicating that the drug acts directly on astrocytes to block neuroinflammation [31]. Like S1P, FTY720-phosphate protects cultured neurons against Aβ toxicity [32], and administration of FTY720 protects against hippocampal neuronal loss in rats given intra-cranial injections of Aβ peptide [33]. FTY720 treatment inhibited Aβ secretion by cultured neurons and reduced Aβ40 levels in the brains of a transgenic mouse model of amyloidosis, but paradoxically promoted Aβ42 accumulation [34].

A single study has demonstrated loss of S1P in cortical tissue from subjects with advanced AD [35], but there is currently no information on whether S1P levels and/or metabolism are affected at earlier stages of AD pathogenesis. Given the importance of S1P in hippocampal synaptic integrity, its association with ApoE, and recent evidence indicating that FTY720 administration can protect against neuronal atrophy in rats administered intra-cranial Aβ, this is clearly an important question to resolve. In the current work, we used a cohort of donor brain tissues that were scored post-mortem for NFT pathology and neuritic plaques according to the staging scheme of Braak and Braak [6,36], and the NIH-Reagan criteria [37]. We demonstrate that loss of S1P proceeds in tandem with the development of NFT pathology, coupled to a decline in the activity of sphingosine kinases, which catalyse S1P synthesis. In particular, hippocampal S1P levels were directly correlated with SphK1 activity, which is known to play an important role in hippocampal synaptic integrity. Lastly, we demonstrate that S1P levels in the hippocampus are independent of Aβ levels, but appear to be associated with APOE genotype as well as Braak NFT stage.

## Methods

### Human brain tissues

Human brain tissue samples were obtained from the New South Wales Tissue Resource Centre and the Sydney Brain Bank. Ethics approval for the current study was from the University of New South Wales Human Research Ethics Committee (HREA11038). Frozen tissue samples were taken from the CA1 region of the hippocampus, inferior temporal gyrus grey and white matter, superior frontal gyrus grey and white matter, and cerebellum, for each of the 34 subjects. Brain tissue was pulverised over dry ice and stored as a powder at -80°C until required for analysis. We have recently reported the age, gender, cause of death, tissue pH, Braak stage, and APOE genotype of the 34 subjects in this cohort [38]. This information is included as Additional file 1: Table S1, together with CERAD scores for neuritic plaque density [37], and concentrations of Aβ40 and Aβ42 in the hippocampus tissue samples, measured using an ELISA, as described below. All subjects in the Braak V/VI group fulfilled NIH-Reagan criteria for a post-mortem diagnosis of AD [37].

### APP_swe_/PS1_ΔE9_ mice

Female double transgenic mice expressing chimeric mouse/human APP with Swedish familial mutations (K595N/M596L) and mutant human Presenilin-1 (*PS1*/*ΔE9*) were obtained from Jackson Laboratory [Bar Harbor, USA; strain name: B6C3-Tg(*APP*_*Swe*_,*PSEN1dE9*)85Dbo/Mmjax; stock no. 004462]. This female transgenic mouse model was compared to genetic background strain C57BL/6. J20 (APP_SwInd_) mice were also obtained from Jackson Laboratory and compared to C57BL/6 controls. Use of these mice was approved by the University of Wollongong animal ethics committee (approval AE11/03).

### Quantification of Aβ in brain tissue

Aβ40 and Aβ42 were quantified in TBS/NP40-soluble and guanidinium-HCl-soluble fractions from human or mouse tissue homogenates as described previously [38], using Beta Amyloid x-40 and x-42 ELISA kits (Covance).

### Lipid extraction and analysis by Liquid Chromatography-Tandem Mass Spectrometry (LC-MS/MS)

Lipids were extracted from 10–15 mg of frozen brain tissue, as described [39], including 50 pmoles each of C17 (d18:1/17:0) Ceramide, C17 (d17:1) S1P and C17 (d17:1) sphingosine as internal standards for mass spectrometry. Lipid extracts were reconstituted in 400 μl of HPLC mobile phase (1 mM ammonium formate in 80% methanol/20% MilliQ water, containing 0.2% formic acid) and stored at -20°C until LC-MS/MS analysis. Ceramide (Cer), S1P and sphingosine quantification was performed on a ThermoFisher Scientific Quantum Access triple quadrupole mass spectrometer equipped with an Accela UPLC and a 3 × 150 mm Agilent XDB-C8 column (5 μm pore size), as described previously [39]. Ceramide (d18:1/14:0, d18:1/16:0, d18:1/17:0, d18:1/18:0, d18:1/20:0, d18:1/22:0, d18:1/24:0, and d18:1/24:1), S1P (d18:1), sphingosine (d18:1), C17 S1P, and C17 sphingosine were analysed simultaneously in multiple reaction monitoring mode. Instrument conditions were optimised prior to analysis using commercially available standards. Transitions monitored for sphingosine, S1P, and ceramides were as described previously [39]. Transitions for C17 sphingosine and C17 S1P were *m/z* 286.1 to 268.0, and 366.1 to 250.1, respectively. The scan time for each event was 0.4 seconds. S1P and sphingosine were quantified as ratios to their C17 internal standard, relative to external calibration curves. Peaks were integrated using Xcalibur software (ThermoFisher Scientific). Analytical (HPLC) grade solvents were purchased from Merck. All lipid standards were purchased from Avanti Polar Lipids (Alabaster, Alabama).

### Protein extraction for enzyme assays and western blots

Tissue extracts (20 mg) were resuspended in 500 μl lysis buffer containing 20 mM Hepes, pH 7.4, 10 mM KCl, 1 mM dithiothreitol, 3 mM β-glycerophosphate and complete protease inhibitor cocktail (Roche, Mannheim, Germany). Extracts were lysed by ultrasonication for 5 min on ice, then cleared by centrifugation at 1000 g for 10 min. Supernatant was stored at -80°C. Protein concentrations were determined with the Bicinchoninic acid (BCA) assay (Thermo Scientific, Rockford, IL, USA).

### Sphingosine kinase assays

SphK1 and SphK2 activity assays were modified from a previous publication [40] to measure formation of C17 S1P from C17 sphingosine, using LC-MS/MS. SphK1 activity was measured in 50 mM Hepes, pH 7.4, 15 mM MgCl_2_, 0.5% Triton X-100, 2 mM ATP, 5 mM NaF, 10 μM fumonison B1, 1 mM 4-deoxypyridoxine, 0.1% BSA; and Sphk2 activity in 50 mM Hepes, pH 7.4, 15 mM MgCl_2_, 0.5 M KCl, 2 mM ATP, 5 mM NaF, 10 μM fumonison B1, 1 mM 4-deoxypyridoxine and 0.1% BSA. To each 50 μL kinase activity assay, 10 μM C17 Sph was added as substrate. Reactions were started with the addition of 5 μg of protein lysate, and the reaction mix was incubated at 37°C overnight. Reactions were stopped with 200 μL of methanol containing 100 pmoles d18:0 dihydrosphingosine 1-phosphate (dhS1P) as the internal standard for LC-MS/MS. Reaction mix was centrifuged to clear insoluble debris (16,000 g, 10 min), and supernatants were transferred to HPLC vials with 300 μl glass inserts for quantification of C17 S1P by LC-MS/MS, as described below.

### S1P phosphatase assay

Total phosphatase activity was modified from a published assay [41], replacing the fluorescently labelled NBD-S1P used with the more physiologically similar C17 S1P. Total phosphatase activity was measured in 1 mM EDTA and 0.1% BSA, to which was added 10 μM C17 S1P as substrate, and 3 μg of protein lysate. Reaction mix (50 μL) was incubated at 37°C for 20 min, and reactions were stopped with the addition of 200 μL methanol containing 100 pmoles d18:0 dihydrosphingosine (dhSph) as the internal standard for LC-MS/MS. The C17 sphingosine formed in the reaction was quantified by LC-MS/MS, as described below.

### Quantification of C17 S1P and C17 sphingosine reaction products by LC-MS/MS

C17 S1P and C17 Sph were quantified using a ThermoFisher Scientific Quantum Access triple quadrupole mass spectrometer, operated in positive ion mode, coupled to a 2.1 × 100 mm Agilent Eclipse Plus C8 column (1.8 μm pore size). Total HPLC time was 7 min per sample at a flow rate of 0.2 mL/min, using the following gradient: 0 min, 20:80 A/B; 3.75 min, 13.5:86.5 A/B; 6.5 min, 13.5:86.5 A/B; and 7 min, 20:80 A/B. Solvent A: 0.2% formic acid, 2 mM ammonium formate in MilliQ water; Solvent B: 0.2% formic acid, 1 mM ammonium formate in methanol. C17 S1P, C17 Sph, dhS1P (d18:0) and dhSph (d18:0) were analysed in multiple reaction monitoring mode, scanning for the following transitions: *m/z* 286.1 to 268.0 (C17 Sph), 302.5 to 284.1 (dhSph), 366.1 to 250.1 (C17 S1P), and 382.2 to 284.2 (dhS1P). The scan time for each event was 0.35 seconds. C17 S1P and C17 sphingosine were quantified as ratios to their respective internal standard (dhS1P or dhSph), using external calibration curves.

### Western blotting

Samples (10 μg) were loaded onto 4-12% Bis-Tris gels (Life technologies, Gaithersburg, MD) and proteins separated by reducing 1-D polyacrylamide gel electrophoresis (1-D SDS-PAGE), as described previously [42], then transferred to a polyvinylidene difluoride (PVDF) membrane for 90 min at 590 mA in carbonate buffer (0.5 M NaHCO_3_, 0.15 M Na_2_CO_3_, 20% v/v methanol) [43]. Membranes were blocked with 5% skim milk in Tris-buffered saline (50 mM Tris, 150 mM NaCl, pH 7.6) containing 0.1% Tween-20 (TBS-T) for 1h at room temperature, then probed with rabbit monoclonal anti-SphK1 (1:1000, #12071, Cell Signalling, Beverly, MA), Sgpp1 (1:500, ab129253, Abcam, Cambridge, UK), Sgpp2 (1:500, Sc-134030, Santa Cruz Biotechnology, Dallas, Texas, USA), or β-actin (1:5000, ab8227, Abcam) at 4°C overnight. Blots were then probed with secondary anti-rabbit IgG, horseradish peroxidise-linked antibody (1:3000, #7074S, Cell Signalling). Antibody-antigen binding was detected using Western Lightning Chemiluminescence Reagent Plus (PerkinElmer, Wellesley, MA) and imaged on a Fujifilm Las-4000 CCD camera (Fujifilm Global, Tokyo, Japan). Protein extract from a single frontal cortex tissue sample was included on every gel as a loading control (LC), to account for any variation in transfer and exposure times during the western blotting process. Bands were quantified by densitometry with Fuji ImageQuant software.

### Statistical analysis

Differences between the control and experimental groups for metabolite levels (S1P, sphingosine, and ceramide), enzyme activity, and protein levels were tested by one-way ANOVA followed by Dunnett’s post-test to compare individual groups to the control. Values were log-transformed for normality prior to testing. One subject was excluded as an outlier in the S1P ANOVA as their S1P/sphingosine ratio was more than 20 standard deviations outside the mean of the entire cohort. Univariate correlations between S1P/sphingosine ratio and enzyme activities or Aβ concentrations were tested by Spearman correlation analysis. These analyses were performed with GraphPad PRISM software (San Diego, CA).

Univariate and multivariate linear regression were used to test associations between the S1P/sphingosine ratio and Braak Stage, APOE genotype, Aβ levels, subject age, and post-mortem interval. The S1P/sphingosine ratio variable was log-transformed for normality. The same outlier from the S1P ANOVA was excluded in these analyses. Significance was established at *p* < 0.05. These analyses were carried out with SAS v9.3 (Cary, NC).

## Results

### S1P levels decline in a regiospecific manner during AD pathogenesis

**Figure 1:**
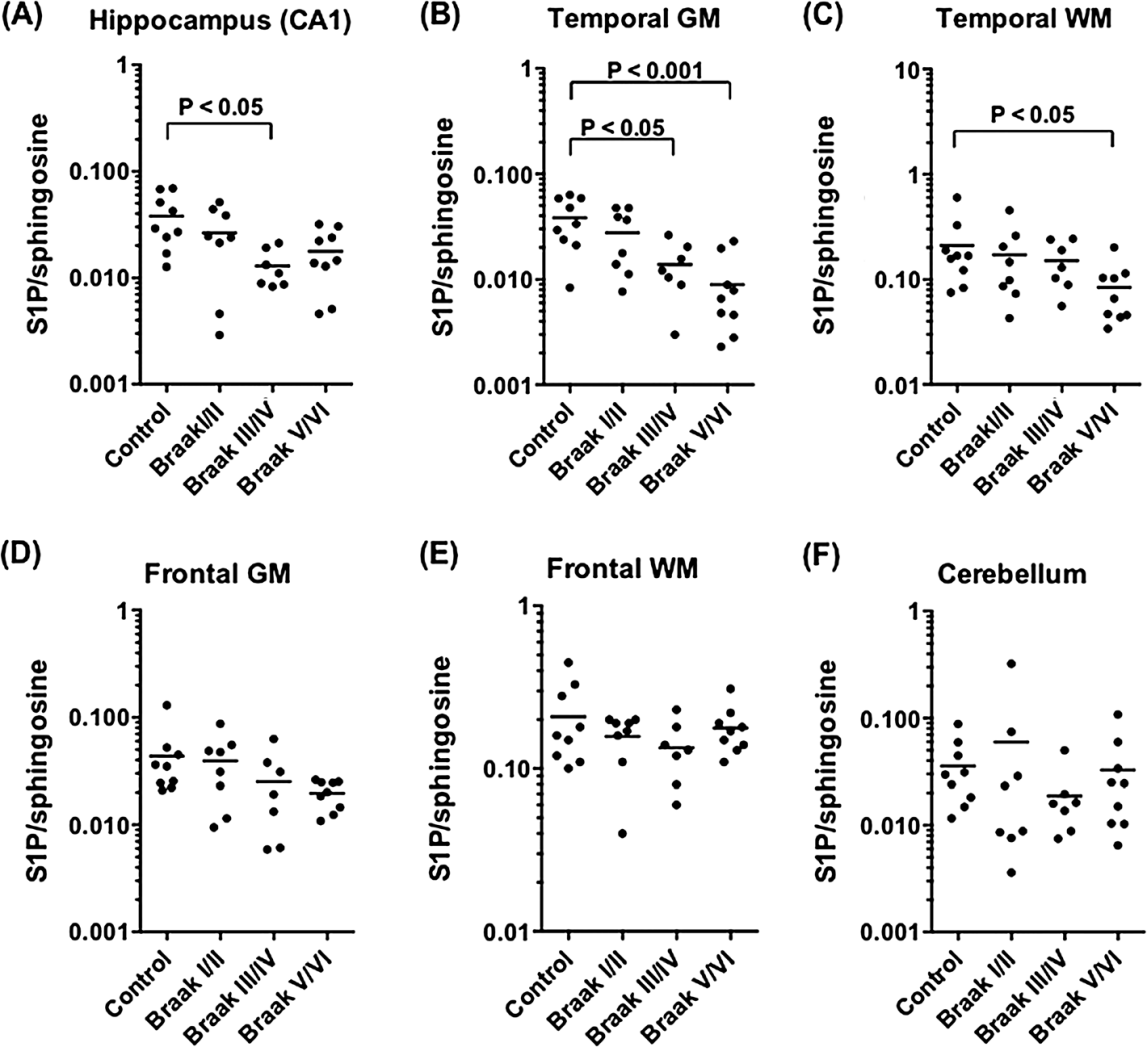
**S1P levels decline during AD pathogenesis. (A – F)** S1P levels, expressed as a ratio to total sphingosine, in human hippocampus **(A)**, inferior temporal GM **(B)**, inferior temporal WM **(C)**, superior frontal GM **(D)**, superior frontal WM **(E)**, and cerebellum **(F)** tissue samples. Samples were divided into four groups based on Braak NFT pathology, as detailed in Results text. Horizontal bars indicate the mean. Statistical significance was determined by a one-way ANOVA, followed by Dunnett’s post test, as described in Methods.

LC-MS/MS was used to examine the spatiotemporal changes to S1P in the course of AD progression. Post-mortem brains were classified into four groups according to the Braak NFT staging system [6,36]: Braak stage I/II, NFT pathology restricted to the entorhinal region (n = 8); Braak III/IV, pathology in the hippocampus and extending into associated cortical regions (n = 7); Braak V/VI, extensive cortical pathology and clinical AD (n = 10); and age-matched controls with no NFT pathology (n = 9). Tissue samples were derived from four brain regions that are differentially affected by NFT pathology during the course of AD pathogenesis: hippocampus (CA1 region) > inferior temporal gyrus > superior frontal gyrus > cerebellum. Both Grey Matter (GM) and White Matter (WM) were analysed for the inferior temporal and superior frontal gyri. S1P content in individual tissue samples was normalized relative to its non-phosphorylated precursor, sphingosine. The normalised S1P level declined with increasing Braak stage, and this change was most pronounced in brain regions where NFT pathology commences earlier in AD pathogenesis. In the hippocampus, mean normalised S1P levels declined by 30% in the Braak I/II group, 66% in the Braak III/IV group (p < 0.05), and 53% in the Braak V/VI group, relative to the control group (Figure 1A). The overall ANOVA result was not significant for the hippocampus (p = 0.055), but became significant when adjusted for APOE genotype, as described in more detail below. In temporal GM, the overall association between normalised S1P level and Braak stage was highly significant (p = 0.0004). Mean normalised S1P levels declined 28% in the Braak I/II group, 64% in the Braak III/IV group (p < 0.05), and 77% in the Braak V/VI group (p < 0.001), relative to the control group (Figure 1B). S1P also declined as a function of Braak stage in temporal WM (Figure 1C), with the mean level reduced by 60% in the Braak V/VI group compared to the control group (p < 0.05). A decline in normalised S1P level relative to Braak stage, although not statistically significant, was also observed in frontal GM (Figure 1D). S1P levels exhibited no significant change relative to NFT pathogenesis in the frontal WM and cerebellum, the latter of which is relatively unaffected in AD (Figure 1E and F). Normalising S1P to tissue mass yielded very similar results, however the sample-to-sample variability was greater (Additional file 1: Figure S1A and B). Sphingosine levels appeared to increase modestly (Additional file 1: Figure S1C and D), suggesting that the balance between sphingosine phosphorylation and dephosphorylation is affected in AD pathogenesis.

### Ceramide levels are not significantly altered in hippocampus and temporal GM

**Figure 2:**
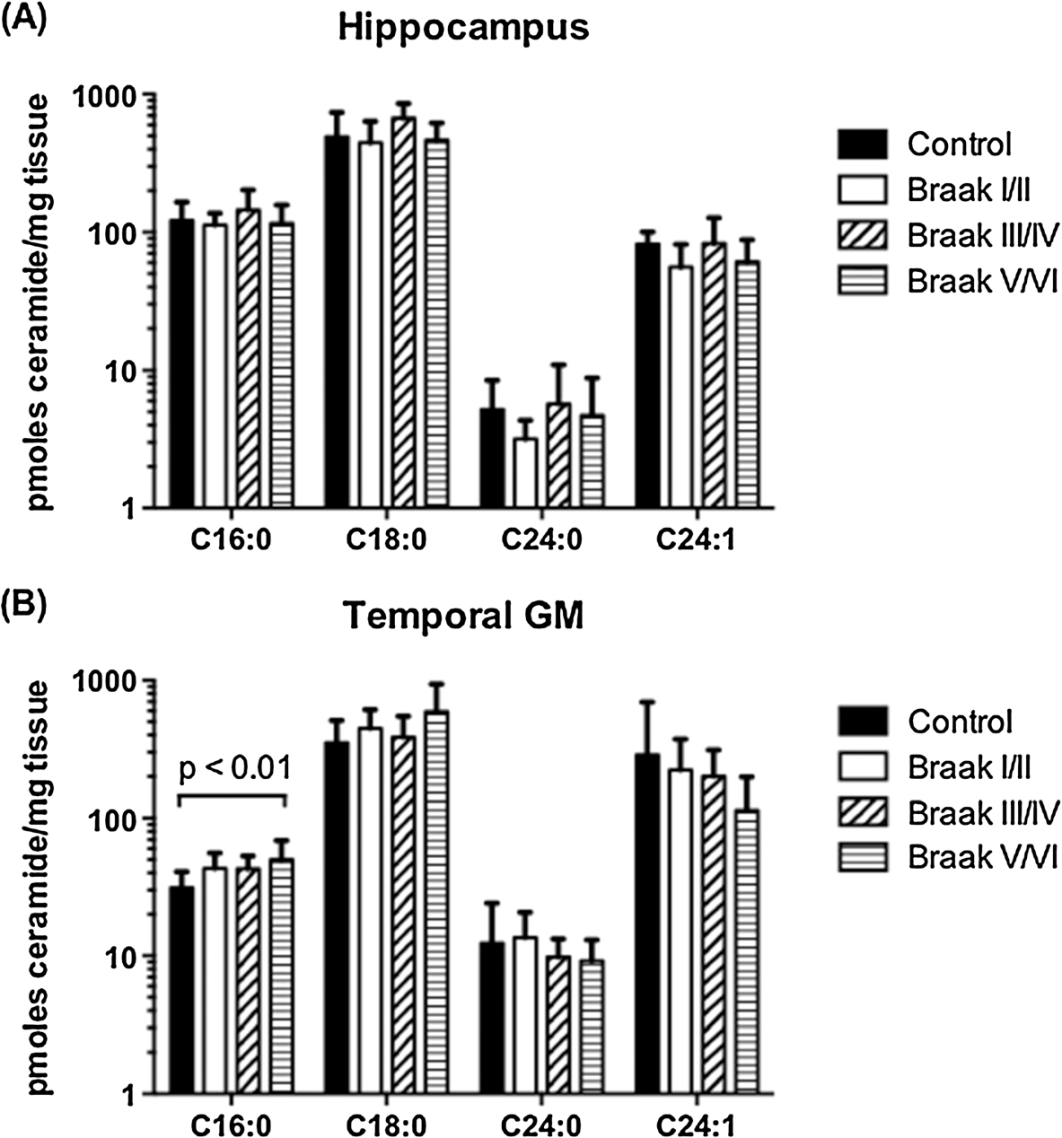
**Ceramide levels in hippocampus and temporal GM remain relatively constant.** Levels of the four most abundant ceramide species in **(A)** hippocampus and **(B)** temporal GM were determined by LC-MS/MS. Ceramide content is expressed relative to tissue mass. Mean and standard deviation for each of the Braak stage groups are shown. Statistical significance was determined by one-way ANOVA, followed by Dunnett’s post test to compare different Braak groupings to the control group.

Sphingosine is formed by deacylation of the central sphingolipid ceramide. Ceramide levels have been reported to increase in several studies using cortical tissues from AD subjects [35,44,45]. Ceramide levels in the hippocampus and temporal GM tissues were quantified, to determine if altered conversion of sphingosine to S1P was accompanied by significant changes to the upstream lipid ceramide (Figure 2). The four most abundant ceramide species (C16, C18, C24, and C24:1) are shown. There were no notable changes to ceramide levels in the hippocampus across the groups. In temporal GM, C16 ceramide levels increased with increasing Braak stage (p = 0.013), but total ceramide content was not significantly different amongst the groups (Additional file 1: Figure S2).

### Altered S1P/sphingosine ratio reflects loss of sphingosine kinase activity

**Figure 3:**
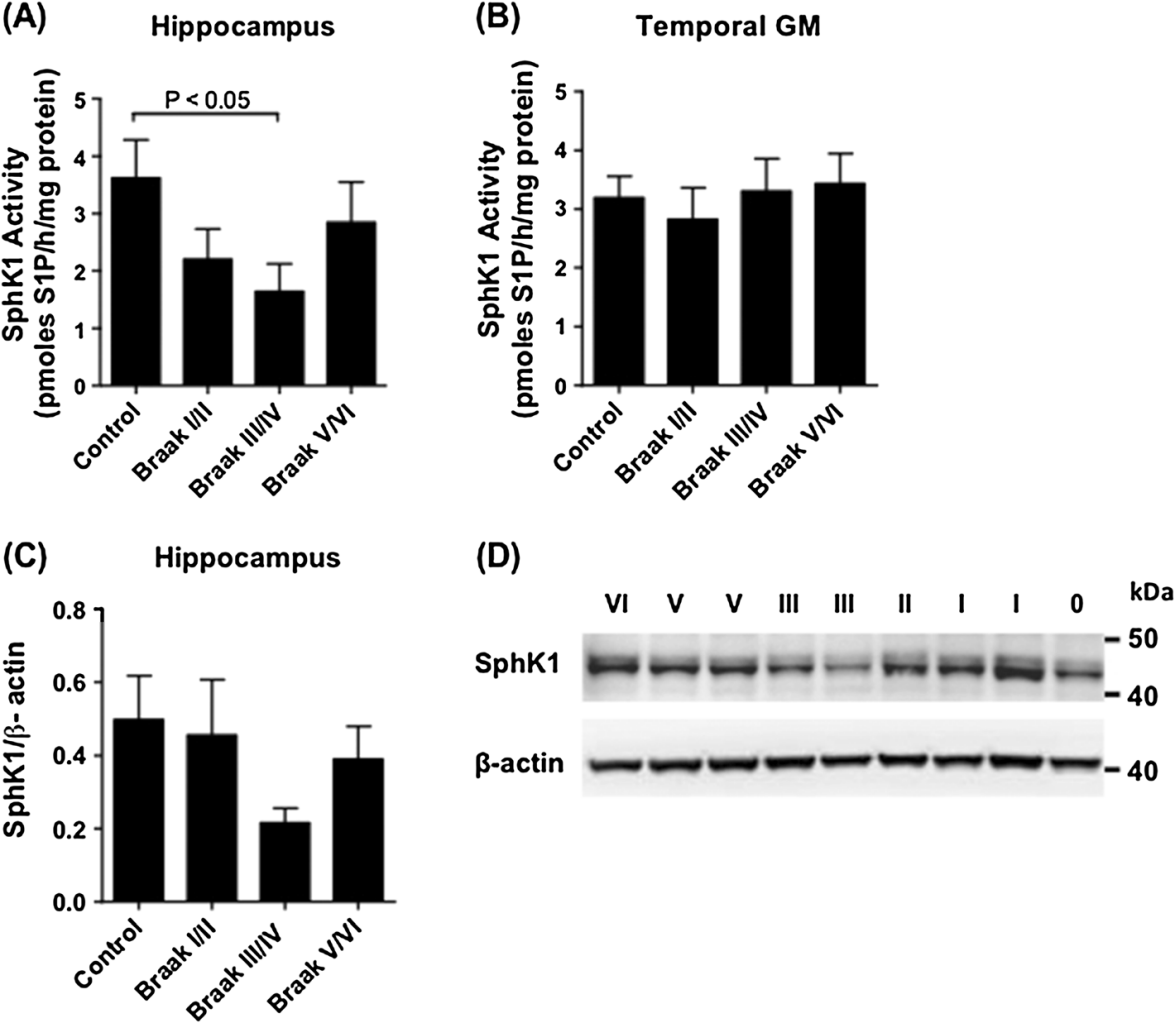
**Reduced SphK1 activity in the hippocampus of Braak III/IV subjects.** SphK1 activity in hippocampus **(A)** and temporal GM **(B)** tissue extracts of control (n = 9), Braak stage I/II (n = 8), Braak III/IV (n = 7), and Braak V/VI (n = 10) brains. Results shown are the mean and standard error for combined results derived from two **(B)** or three **(A)** independent assays. **(C)** SphK1 protein levels in the hippocampus were determined by western blotting and normalised to housekeeping gene β-actin. **(D)** Example western blot showing SphK1 protein in hippocampus tissue samples. Braak stage is indicated for each sample. Statistical significance was determined using a one-way ANOVA and Dunnett’s post test.

**Figure 4:**
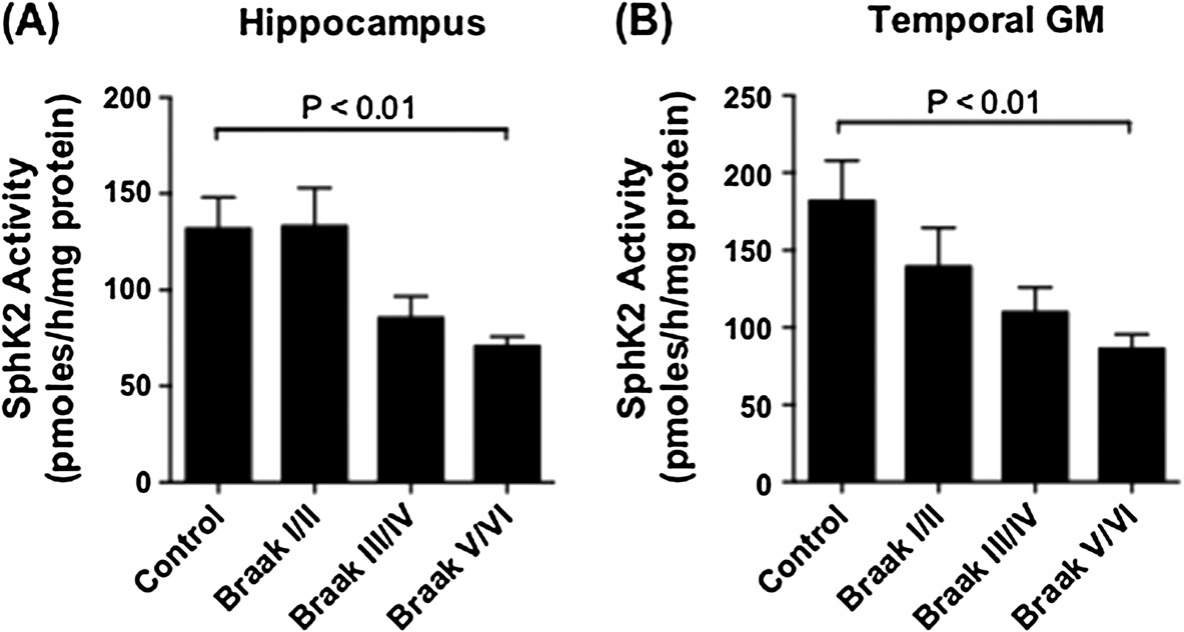
**SphK2 activity declines during AD pathogenesis.** SphK2 activity in hippocampus **(A)** and temporal GM **(B)** tissue extracts of control (n = 9), Braak stage I/II (n = 8), Braak III/IV (n = 7), and Braak V/VI (n = 10) brains. SphK2 activity was assayed as described in methods. Results shown are mean and standard error for combined results from two independent enzyme activity assays. Statistical significance was determined using a one-way ANOVA and Dunnett’s post test.

The shift in S1P/sphingosine equilibrium in the hippocampus and temporal cortex prompted us to measure the activity of SphK1 and SphK2, which catalyse sphingosine phosphorylation; as well as S1P phosphatase activity, which converts S1P back to sphingosine. SphK1 and 2 activity were assayed by measuring the phosphorylation of 17-carbon (C17) sphingosine (naturally occurring sphingosine is 18 carbons in length) by crude tissue homogenates, in the presence of 0.5% TritonX-100 (which favours SphK1 and inhibits SphK2) or 0.5 M KCl (which favours SphK2 activity and inhibits SphK1) [40,46]. SphK1 activity declined with increasing Braak stage in the hippocampus (p = 0.032 by One-Way ANOVA), although mean SphK1 activity rebounded to some extent in the Braak V/VI group (Figure 3A). In contrast to the hippocampus, SphK1 activity did not differ with Braak stage in temporal GM (Figure 3B). The loss of SphK1 activity in Braak III/IV hippocampus samples was accompanied by a decline in levels of the protein, although not statistically significant (Figure 3C and D). SphK2 activity declined significantly with increasing Braak stage in both the hippocampus and temporal GM (p = 0.0009 and 0.0098 by ANOVA, respectively) (Figure 4A and B). We were unable to confidently identify SphK2 protein by western blotting on our brain extracts, using primary antibodies from a number of different suppliers.

**Figure 5:**
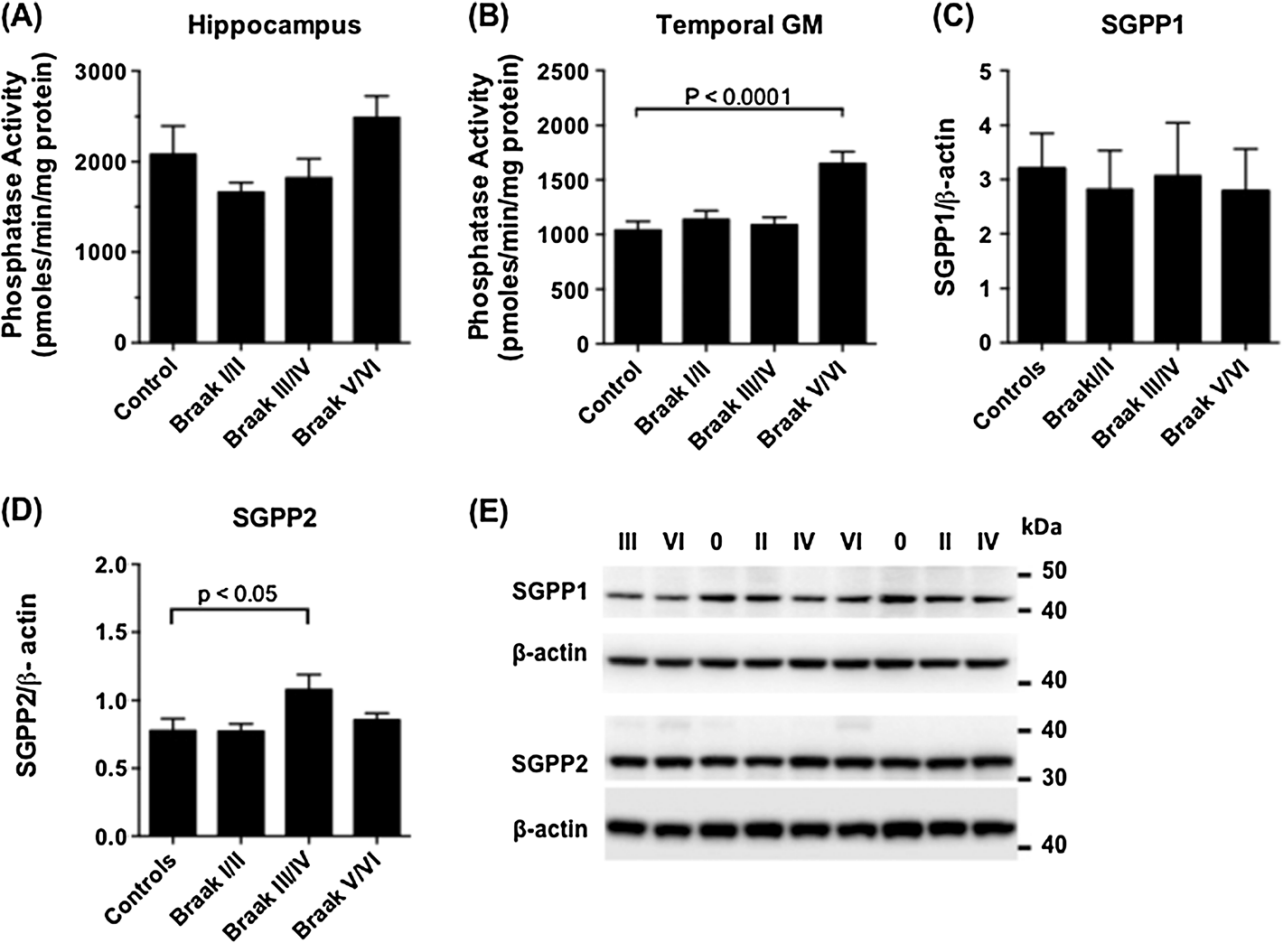
**S1P phosphatase activity increases in temporal GM of AD brains.** Total S1P phosphatase activity in hippocampus **(A)** and temporal GM **(B)** extracts of control (n = 9), Braak stage I/II (n = 8), Braak III/IV (n = 7), and Braak V/VI (n = 10) brains. Overall association between S1P phosphatase activity activity and Braak stage was significant (P < 0.0001 by one-way ANOVA) for temporal GM but not hippocampus (P = 0.063). Sgpp1 **(C)** and Sgpp2 **(D)** protein levels in the temporal GM tissue extracts were determined by western blotting and normalised to housekeeping gene β-actin. **(E)** Example western blots for Sgpp1 and Sgpp2 protein. Braak stage is indicated for each sample.

Total S1P phosphatase activity was measured as the capacity for whole tissue homogenates to catalyse the dephosphorylation of 17-carbon (C17) S1P. S1P phosphatase activity was relatively unchanged at the different Braak stages in the hippocampus (Figure 5A), but was 59% higher in the temporal GM of Braak V/VI subjects compared to the controls (p < 0.0001) (Figure 5B). Sphingosine 1-Phosphate Phosphatases 1 and 2 (Sgpp1 and 2) specifically catalyse the dephosphorylation of S1P [47,48]. There was no increase in the protein levels of either enzyme in the temporal GM of the Braak V/VI group that would account for the robust increase in S1P phosphatase activity (Figure 5C - E).

SphK1 but not SphK2 or S1P phosphatase activity was positively correlated with normalised S1P level in the hippocampus samples (r_s_ = 0.43, p = 0.012), strongly suggesting that declining S1P/sphingosine is directly related to loss of SphK1 activity in the hippocampus. Despite declining SphK2 activity with increasing NFT pathology, none of SphK1, SphK2, or S1P phosphatase activity were significantly correlated with S1P/sphingosine in temporal GM.

### Aβ overproduction in mice does not cause a reduction in normalised S1P

Concentrations of the amyloidogenic 40 and 42 amino acid Aβ peptides (i.e. Aβ40 and Aβ42) in the human hippocampus tissue samples used in this study are shown in Additional file 1: Table S1. Mean values for the four Braak staging groups have been reported previously [38]. Soluble and insoluble Aβ42 peptide levels increased dramatically in the Braak stage V/VI cohort, but did not increase appreciably in earlier stage subjects (Additional file 1: Table S1). Correlation analysis indicated that there was no association between soluble or insoluble Aβ42 peptide levels and the normalised S1P level in the hippocampus (p = 0.18 and 0.21, respectively).

**Figure 6:**
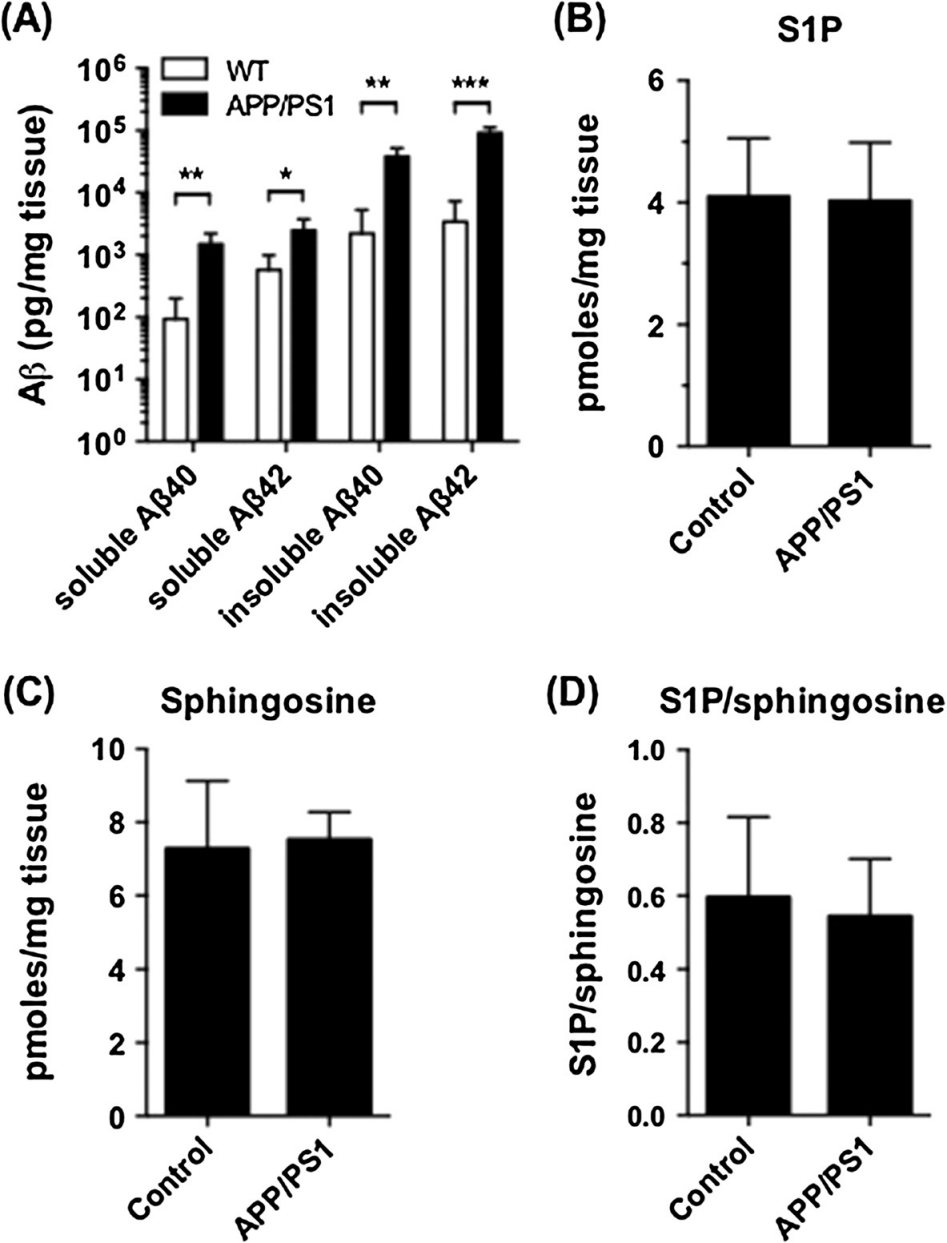
**Aβ overproduction in mice does not cause a reduction in normalised S1P.** Soluble and insoluble Aβ40 and Aβ42 **(A)**, S1P **(B)**, and sphingosine **(C)**, were quantified in cortical tissue from APP_swe_/PS1_ΔE9_ (n = 4) or control C57BL/6 (n = 4) mice. S1P/sphingosine ratio is shown in **(D)**. Mean and standard deviation are shown. Statistical significance of differences between the two sample groups was tested with t-tests, adjusted for multiple comparisons: *p < 0.05; **p < 0.01; ***p < 0.001.

To test whether Aβ overproduction causes a reduction in S1P levels and/or the S1P/sphingosine ratio, S1P and sphingosine were quantified in cortical tissue from 11 month old APP_swe_/PS1_ΔE9_ mice. These mice are characterised by familial mutations in APP and presenilin 1 that give rise to accelerated Aβ deposition in the brain [49] (Figure 6A). Levels of S1P and sphingosine were unchanged between APP_swe_/PS1_ΔE9_ and control C57BL6 mice (Figure 6B - D). There was also no reduction in the brain S1P/sphingosine ratio in 16 month old J20 (APP_SwInd_) transgenic mice, which also accumulate Aβ plaques [50] (data not shown).

### S1P/sphingosine is significantly associated with APOE genotype in the hippocampus

**Figure 7:**
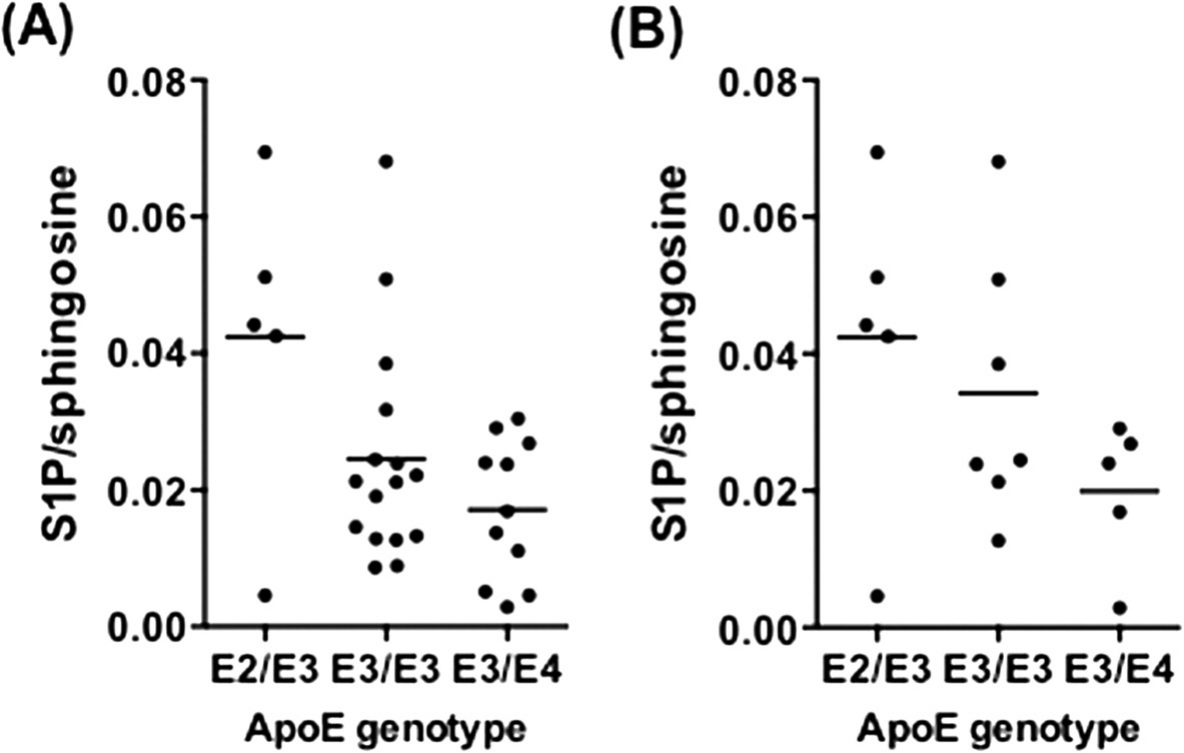
**Hippocampal S1P level is related to APOE genotype. (A)** S1P/sphingosine ratio in the hippocampus of subjects grouped according to APOE genotype: ϵ2/ϵ3 (n = 5), ϵ3/ϵ3 (n = 16), ϵ3/ϵ4 (n = 11). **(B)** S1P/sphingosine data after removing subjects with Braak III-VI pathology: ϵ2/ϵ3 (n = 5), ϵ3/ϵ3 (n = 7), ϵ3/ϵ4 (n = 5). Horizontal bars indicate the mean.

We have also previously published the APOE genotype of subjects for this cohort [38], and show herein that the normalised S1P level is 2.5-fold higher in the hippocampus of APOE ϵ2 carriers, compared to ϵ4 carriers (Figure 7A). The influence of APOE genotype cannot easily be differentiated from the effect of Alzheimer’s pathology in this sample set. However, after removing subjects with significant hippocampal NFT pathology (Braak stages III – VI), the overall trend was maintained (Figure 7B), raising the possibility that APOE genotype influences S1P/sphingosine balance before the development of NFT pathology in the hippocampus. We therefore constructed a multivariate linear regression model to test how NFT pathology (i.e. Braak stage) and APOE genotype are associated with the S1P/sphingosine ratio in the hippocampus (Table 1). In this model, both Braak stage and APOE genotype were significantly associated with normalised S1P (p = 0.039 and 0.0495 respectively). Hippocampus was the only brain region in which a relationship between normalised S1P level and APOE genotype was apparent (data not shown for other brain regions). Accordingly, modelling interactions between normalised S1P level, APOE genotype, and Braak stage in the temporal cortex GM indicated that Braak stage but not APOE genotype is significantly associated with normalised S1P levels (p = 0.002 and 0.995 respectively).

**Table 1:** Multivariate regression estimates and associated p-values for normalised S1P in the hippocampus

**Variable**	***Coefficient (β)***^***1***^	***SE B***^***2***^	***95% CI***	***p-value***
Braak Stage				0.0392
Ctrl vs Braak I/II	-0.443	0.35	-1.545, -0.090	0.0290
Ctrl vs Braak III/IV	-0.533	0.39	-1.900, -0.283	0.0101
Ctrl vs Braak V/VI	-0.393	0.34	-1.400, 0.002	0.0506
APOE Genotype				0.0495
ϵ3/ϵ3 vs ϵ2/ϵ3	0.092	0.39	-0.605, 1.013	0.6090
ϵ3/ϵ3 vs ϵ3/ϵ4	-0.403	0.29	-1.282, -0.074	0.0292
*R*^*2*^		0.37		
*F*		3.12		0.025

Reference group for Braak Stage is Control; reference group for APOE Genotype is ϵ3/ϵ3.

^1^Standardized regression coefficient; ^2^Standard error of the coefficient.

## Discussion

In this study, we demonstrate for the first time that levels of the potent lipid signalling metabolite S1P decline in a regiospecific manner during the course of AD pathogenesis. This loss of S1P tracked with Braak pathology, being most apparent in brain regions that are affected relatively early in AD pathogenesis, and absent in the cerebellum, which is spared in AD. There was a statistically significant decline in the level of S1P, when normalised to its non-phosphorylated precursor sphingosine, in the hippocampus, temporal GM, and temporal WM with increasing NFT pathology (Figure 1). S1P levels were normalised relative to sphingosine to offset the high sample-to-sample variability that is characteristic of human tissue studies and confounds statistical analyses. This approach is in accordance with the accepted approach for signalling proteins, whereby phosphoprotein levels are normalised to the total amount of the particular protein being measured. The results were very similar, although variances were larger, when S1P content was normalised to tissue mass, and the decline in this measure was statistically significant in temporal GM of Braak V/VI subjects (Additional file 1: Figure S1). Our results with the Braak V/VI subjects, all of whom had been diagnosed with AD, are in agreement with a previous study demonstrating a significant loss of S1P in frontotemporal GM from subjects with clinical AD [35]. However, our study goes much further, firstly by demonstrating loss of S1P prior to AD diagnosis, secondly by demonstrating that it occurs in a regiospecific manner, and thirdly by attributing loss of S1P to a decline in sphingosine kinase activity.

He *et al.* reported a strong inverse correlation between tissue Aβ levels, as measured by ELISA, and S1P [35], but this was not observed in our study. An important difference is that our study was primarily concerned with changes that occur prior to clinical or neuropathological diagnosis of AD, and therefore included tissue from subjects that showed Braak stage III/IV pathology but only rare neuritic plaques and low Aβ concentrations (Additional file 1: Table S1) [37]. Supporting the mechanistic disconnect between Aβ levels and S1P loss, overproduction of Aβ in the APP_swe_/PS1_ΔE9_ transgenic mouse model did not drive S1P levels down. These mouse brains were assessed at an age at which significant amyloid production and plaque deposition has occurred. Whilst loss of S1P in our study cannot be directly correlated to Aβ levels measured by ELISA, previous publications have demonstrated that S1P and its synthetic analogue FTY720-phosphate confer protection against loss of neuron viability induced with Aβ [23,32]. On this basis, we speculate that regiospecific loss of S1P may sensitize those brain regions that are affected earlier in AD to Aβ toxicity.

S1P is derived in two enzymatic steps from the more abundant lipid ceramide, whose levels have been reported as increased in a number of studies using frontal cortex tissue from AD subjects [35,44,45]. Increased production of ceramides may contribute to AD pathogenesis, as ceramide is a pro-apoptotic signalling molecule [51,52]. A modest increase in C16 ceramide was observed in temporal GM with increasing Braak stage (Figure 2), but overall ceramide levels remained relatively constant in the hippocampus and temporal GM of our cohort. To the best of our knowledge, no previous studies have examined ceramide levels in post-mortem hippocampal tissue from subjects with AD, however the lack of major changes to ceramide levels in temporal GM is consistent with a previous study [53]. Increased C16 ceramide in temporal GM may result from loss of SphK2 activity, as has been demonstrated previously [54,55].

SphK1 activity declined with increasing Braak pathology in the hippocampus but not temporal GM (Figure 3), and hippocampal SphK1 activity was significantly correlated with S1P levels, showing a maximal decline in the Braak III/IV group. Loss of hippocampal SphK1 activity was associated with a decline in levels of the protein as detected by western blotting. The rebound in SphK1 activity in the Braak V/VI cohort may reflect astrogliosis, as SphK1 is up-regulated in astrocytes, in response to inflammatory stimuli [56]. SphK1 localises to pre-synaptic terminals in mouse hippocampal neurons where it mediates glutamate release [24] and long-term potentiation [25], leading us to speculate that loss of SphK1 activity in the hippocampus may reflect, and/or contribute significantly to loss of functional synapses. Cholinergic neurons in the basal forebrain are the first to be affected by AD pathology, and in this regard it is interesting to note that cholinergic stimulation of *C. elegans* motor neurons results in translocation of SphK1 to pre-synaptic terminals, where it plays a significant role in neurotransmitter release [26].

SphK2 activity declined with increasing Braak stage in both the hippocampus and temporal cortex (Figure 4), and the declining S1P/sphingosine ratio during AD pathogenesis in the temporal GM appears to reflect reduced SphK2 activity, as well as a robust gain of S1P phosphatase activity in the Braak V/VI brains (Figure 5). SphK2 is believed to be the predominant enzyme responsible for S1P synthesis in the mouse brain [57,58]. In agreement with this, sphingosine kinase activity in our human brain tissue samples was much higher in the presence of high salt, which inhibits SphK1 (Figure 4), than in the presence of TritonX-100, which inhibits SphK2 (Figure 3). Total sphingosine kinase activity in mouse brain tissue was reported to be highest in cerebellum and low in hippocampus, and SphK2 mRNA was significantly higher in cortex than in hippocampus [57]. It is therefore possible that SphK1 is relatively more important for S1P generation in the hippocampus compared to other brain regions. To the best of our knowledge, the anatomical distribution and cell specificity of SphK1 expression throughout the brain has not been reported, although within the mouse hippocampus, SphK1 expression was shown to be highest in mossy fibres [25]. A more extensive investigation of SphK1 and SphK2 expression in the brain, including their relative expression in different cell types, would greatly help in resolving the molecular basis for loss of these enzyme activities during AD pathogenesis. However, the lack of availability of commercial SphK2 antibodies that are well validated for western blotting and immunohistochemistry remains a barrier.

The loss of SphK2 activity in hippocampus and temporal GM observed in our study sits in contrast to the findings of Takasugi *et al.*[59], who reported gain of SphK2 activity in frontal cortex of AD patients. A likely explanation for this difference is that Takasugi *et al.* performed their measurements on Tris-soluble fractions from frontal cortex tissue, whereas our measurements were performed with total homogenate including the membrane fraction. Our results on SphK2 activity are well supported by our lipid measurements. It remains to be determined whether loss of SphK2 activity is related to declining levels of the enzyme, as we were unable to confidently detect and quantify SphK2 protein by western blotting.

Multivariate regression hints at an association between APOE genotype and normalised S1P levels in the hippocampus, although our study was not specifically designed to test this association. The possibility of a direct relationship between APOE genotype and hippocampal S1P levels should be further investigated with a larger cohort of donor tissue samples. The possibility that APOE genotype directly affects brain S1P levels is supported by previous work demonstrating that S1P is secreted from cultured astrocytes into ApoE-containing HDL particles, and that ApoE overexpression in astrocytes promotes S1P secretion [17]. These authors have also shown that S1P is associated with the HDL fraction of cerebrospinal fluid [18]. S1P is an established signalling factor in plasma HDL, whereby the cardioprotective properties of plasma HDL are mediated at least in part via S1P signalling through S1P_3_ receptors [60]. It is likely that S1P associated with HDL particles performs similar cytoprotective functions in the CNS. It would be interesting in future studies to investigate whether there is any change to S1P levels in the cerebrospinal fluid (CSF) of subjects with mild cognitive impairment or AD; and whether S1P levels in the CSF vary with APOE genotype. We note, however, that a pronounced reduction in normalised S1P levels was only observed in brain regions that are heavily affected by AD pathology, and the relationship between APOE genotype and normalised S1P levels was only observed in the hippocampus. These regiospecific alterations may not translate to the CSF.

It is not possible with the current dataset to determine definitively whether loss of sphingosine kinase activity and S1P precedes NFT formation, or vice-versa. Although the two measures are well correlated in the hippocampus and temporal GM, a number of observations suggest that loss of sphingosine kinase activity is not simply a consequence of tangle formation: firstly, S1P/sphingosine ratio and SphK2 activity in temporal GM declined steadily with increasing Braak stage and were clearly apparent in the Braak III/IV group, whilst NFT pathology first appears at Braak stage IV in this brain region. Unfortunately, the sample sizes available for our study were not large enough to examine Braak III and IV groups separately. Secondly, S1P and SphK2 activity in the brain, particularly in grey matter regions, are likely to be primarily astrocyte-derived [17,57]. Therefore, declining SphK2 activity probably reflects astrocyte dysfunction. Nonetheless, feedback from dysfunctional neurons may drive SphK2 activity down in the astrocytes. Thirdly, the potential relationship between hippocampal S1P and APOE genotype suggests a more complex basis for reduced S1P/sphingosine levels in the hippocampus.

## Conclusions

The potent neuroprotective signalling lipid S1P declines in a regiospecific manner during the course of AD pathogenesis, correlating well with the development of NFT pathology in the brain. Declining S1P levels with increasing NFT pathology can be attributed primarily to a loss of both SphK1 and SphK2 activity in the hippocampus, and SphK2 activity in inferior temporal cortex. Loss of S1P in regions such as the hippocampus and temporal cortex may sensitize these regions to synaptic loss and neuronal cell death during the ageing process and/or in response to amyloid accumulation. The S1P signalling system has been the subject of intense pharmacological investigation in recent years, following the discovery and subsequent FDA approval of FTY720 for treatment of relapsing multiple sclerosis. Our findings raise the possibility that pharmacological correction of S1P signalling defects may be applicable in the treatment of AD. Further studies aimed at better defining how altered S1P signalling contributes to AD pathogenesis are clearly warranted.

## Abbreviations

Aβ: Amyloid-β peptide; AD: Alzheimer’s disease; APP: Amyloid precursor protein; CNS: Central nervous system; GM: Grey matter; HDL: High density lipoprotein; LC-MS/MS: Liquid Chromatography-Tandem Mass Spectrometry; NFT: Neurofibrillary tangle; WM: White matter.

## Competing interests

The authors declare that they have no competing interests.

## Authors’ contributions

TAC, NK, and XYL performed experimental work and analysed data; BD performed statistical analysis; CS and JK performed pathological assessment of human tissue samples; RP assisted with mass spectrometry; HL and BG performed amyloid ELISA assays and analysed data; BG and ASD obtained ethics approvals and human tissue samples; TAC, NK, and ASD wrote the manuscript; ASD managed the project. All authors read and approved the final manuscript.

## Supplementary Material

Additional file 1: Table S1 Clinical information for human brain tissue cohort. **Figure S1.** S1P (A and B) and Sphingosine (C and D) levels were quantified in hippocampus (A and C) and temporal GM (B and D) tissue extracts. **Figure S2.** Total ceramide levels were quantified for (A) Hippocampus and (B) Temporal GM tissue extracts by LC-MS/MS. Click here for file
