# Supplementary material for: Loss of the neuroprotective factor Sphingosine 1-phosphate early in Alzheimer’s disease pathogenesis
Source: Acta Neuropathol Commun. 2014 Jan 23;2:9. doi: 10.1186/2051-5960-2-9 (PMC3906863; doi:10.1186/2051-5960-2-9)

Table S1. Clinical information for human brain tissue cohort. Tissue samples were taken from the six different brain regions described in Materials and Methods, for each of the 34 donor brains. Abbreviations: F, Female; M, male; PMI, postmortem interval; Aβ, Amyloid-β; CERAD, Consortium to Establish a Registry for Alzheimer's disease neuritic plaque scoring system [37]. Some of this demographic information has been published previously [38].

Sample ID	Gender	Age	PMI (h)	Cause of Death	Tissue pH	APOE genotype	Braak Stage	CERAD grade[1]	TBS-Soluble Aβ[2]		TBS-Insoluble Aβ[3]	
									$A\beta_{42}$	$A\beta_{40}$	$A\beta_{42}$	$A\beta_{40}$
1	F	85	23	Pneumonia	6.4	3/3	0	0	142	200	95727	238
2	F	102	5	Acute renal failure	5.92	2/3	0	1	11	0.0	15470	57
3	F	84	6	Respiratory arrest	6.5	3/4	0	1	78	193	77125	513
4	M	66	23	Cardiac	6.7	3/4	0	0	14	116	7906	0.0
5	M	68	45	Cardiac	6.1	3/4	0	2	25	231	15638	175
6	M	69	52	Cardiac	7.0	3/3	0	1	20	180	6118	121
7	M	57	18	Cardiac	6.4	3/4	0	1	20	210	2318	179
8	M	64	17	Cardiac	6.6	3/3	0	1	16	158	3314	148
9	M	79	8	Pulmonary embolism	6.7	2/3	0	0	17	199	4879	170
10	F	104	27	Respiratory	5.9	2/3	I	1	6.5	108	5627	184
11	F	78	45	Toxicity	6.1	3/4	I	1	5.0	93	5490	156
12	M	63	24	Cardiac	6.9	3/3	I	1	15	161	3044	169
13	M	62	46	Cardiac	7.0	2/3	I-II	1	90	234	53744	184
14	F	85	10	Respiratory	6.6	3/3	II	1	25	237	2802	175
15	M	69	19	Cardiac	6.3	3/3	II		9.4	130	2167	181
16	M	91	16	Pneumonia	6.5	2/3	II	0	0.0	33	11407	209
17	M	103	20	Myocardial infarct	6.1	3/3	II	0	33	128	57484	251
18	F	81	28	Respiratory	6.2	3/3	III	2	12	161	8149	168
19	F	73	45	Cardiac	6.9	3/3	III	3	20	206	19229	190
20	F	92	14	Cancer	5.6	3/3	III	1	0.7	114	3598	170
21	M	67	25	Cardiac	6.7	3/4	III	2	115	242	117036	638
22	F	83	64	Stroke	6.3	2/4	II-IV	3	99	151	86448	447
23	F	98	6	Cardiac/Respiratory	6.7	3/3	IV	1	6.9	111	4410	161
24	F	92	5	Pancytopaenia	6.1	3/3	IV	1	19	127	12403	155
25	F	94	7	Cardiac arrest	6.1	3/3	V	2	128	234	65083	376
26	F	83	3	Uraemia	5.9	3/3	V	2	66	141	111990	205
27	F	100	3	Aspiration pneumonia	6.4	3/4	V	2	211	227	129147	425
28	F	98	11	Cerebrovascular occlusion	6.1	3/3	V	2	29	167	70089	382
29	F	84	6	Aspiration pneumonia	6.3	3/4	VI	3	259	361	122557	415
30	F	80	32	Cardiorespiratory failure	6.5	3/4	VI	3	323	180	186672	552
31	F	85	10	Cardiorespiratory failure	5.9	3/4	VI	3	21	90	43116	198
32	M	68	23	Cardiorespiratory failure	6.0	3/4	VI	3	270	253	262128	682
33	M	69	3	Colon cancer	6.7	3/3	VI	3	47	215	25530	594
34	M	67	9	Cardiorespiratory failure	6.5	3/3	VI	2	237	292	64634	574

[1] CERAD scoring system for presence of neuritic plaques: 0 (no neutric plaques); 1 (subtle); 2 (moderate); 3 (frequent)

[2] TBS-Soluble amyloid-β levels in the hippocampus, extracted using TBS, values are in pg/mg protein.

[3] TBS-Insoluble amyloid-β levels in the hippocampus, guanidine HCl-extracted, values are in pg/mg protein.

Figure S1. S1P (A and B) and Sphingosine (C and D) levels were quantified in hippocampus (A and C) and temporal GM (B and D) tissue extracts. Sample groupings are detailed in results. Horizontal bars indicate the mean. Statistical significance was determined by a one-way ANOVA, followed by Dunnett's post test, as described in methods.

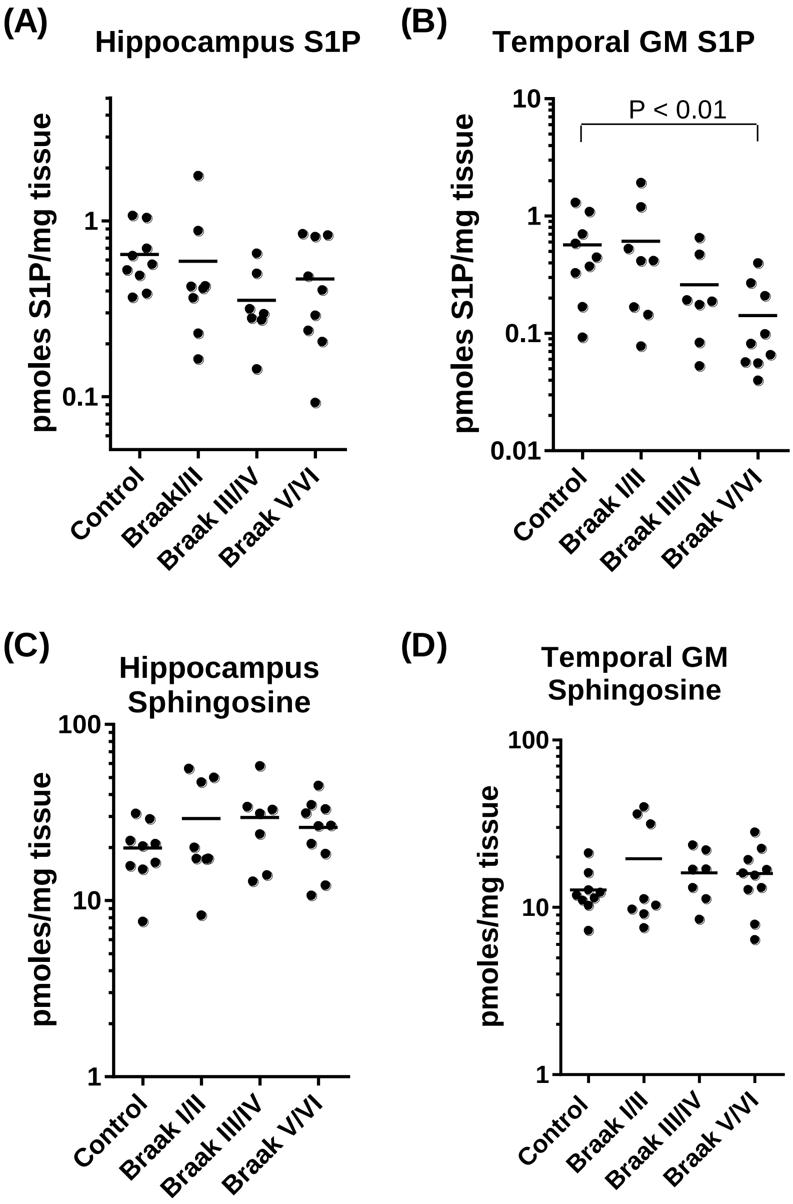

Figure S2. Total ceramide levels were quantified for (A) Hippocampus and (B) Temporal GM tissue extracts by LC-MS/MS. Ceramide content is expressed relative to tissue mass. Horizontal bars indicate the mean. Statistical significance was determined by one-way ANOVA, followed by Dunnett's post test to compare different Braak groupings to the control group. No significant differences were observed.

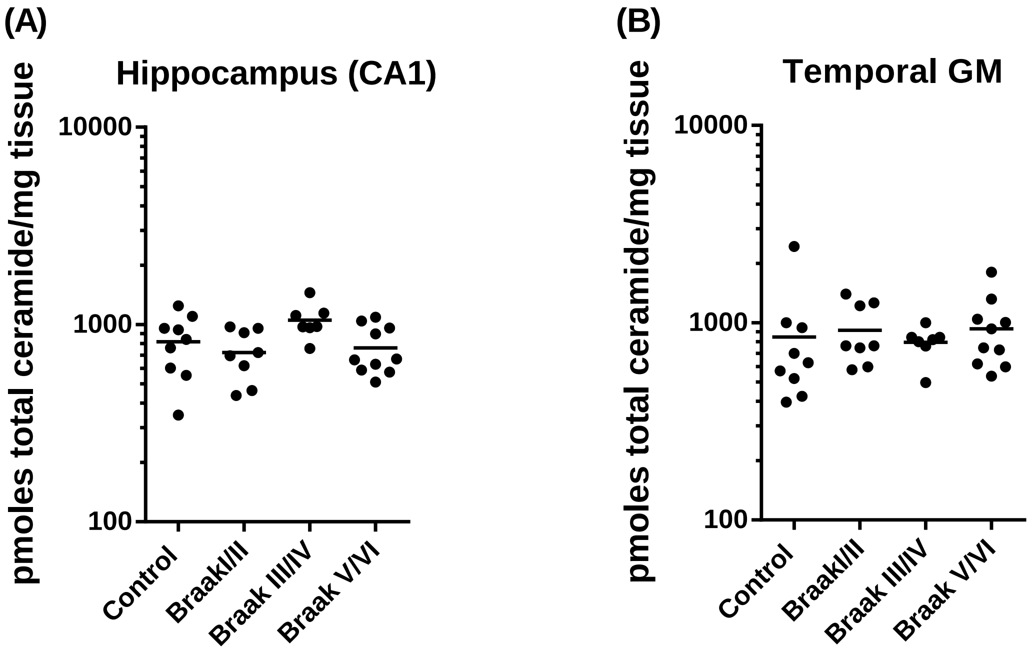

Supplement: Additional file 1: Table S1 — Clinical information for human brain tissue cohort. Figure S1. S1P (A and B) and Sphingosine (C and D) levels were quantified in hippocampus (A and C) and temporal GM (B and D) tissue extracts. Figure S2. Total ceramide levels were quantified for (A) Hippocampus and (B) Temporal GM tissue extracts by LC-MS/MS. [file 2051-5960-2-9-S1.pdf]
